# *In vivo* and *in silico* studies of the effects of oil extracted from *Cannabis sativa* L. seeds on healing of burned skin wounds in rats

**DOI:** 10.3389/fchem.2024.1381527

**Published:** 2024-06-11

**Authors:** Mouna Bouarfa, Mohamed Chebaibi, Fatima Ez-Zahra Amrati, Zouhair Souirti, Hamza Saghrouchni, Yassine El atki, Khalid Bekkouche, Hajar Mourabiti, Amina Bari, John P. Giesy, Mohamed Mohany, Salim S. Al-Rejaie, Mourad A. M. Aboul-Soud, Dalila Bousta

**Affiliations:** ^1^ Laboratory of Biotechnology, Environment, Agri-Food and Health (LBEAS), Faculty of Sciences Dhar El Mehraz, Sidi Mohamed Ben Abdellah University, Fez, Morocco; ^2^ Ministry of Health and Social Protection, Higher Institute of Nursing Professions and Health Techniques, Fez, Morocco; ^3^ Biomedical and Translational Research Laboratory, Faculty of Medicine and Pharmacy of Fez, Sidi Mohamed Ben Abdellah University, Fez, Morocco; ^4^ Clinical Neurosciences Laboratory, Faculty of Medicine and Pharmacy of Fez, Sidi Mohamed Ben Abdellah University, Fez, Morocco; ^5^ Neurology Department, Sleep Center Hassan II University Hospital, Sidi Mohamed Ben Abdellah University, Fez, Morocco; ^6^ Department of Biotechnology, Institute of Natural and Applied Sciences, Çukurova University, Adana, Türkiye; ^7^ High Institute of Nursing Professions and Health Techniques, Fez, Morocco; ^8^ Laboratory of Agri-Food, Biotechnologies and Valorization of Plant Bioresources (AGROBIOVAL), Department of Biology, Team of Protection and Valorization of Plant Resources (AgroBiotech Center, URL-CRNST 05), Faculty of Sciences Semlalia, Cadi Ayyad University, Marrakesh, Morocco; ^9^ Service de Toxico-pharmacologie, Fès, Morocco; ^10^ Department of Veterinary Biomedical Sciences and Toxicology Centre, University of Saskatchewan, Saskatoon, SK, Canada; ^11^ Department of Environmental Sciences, Baylor University, Waco, TX, United States; ^12^ Department of Integrative Biology and Centre for Integrative Toxicology, Michigan State University, East Lansing, MI, United States; ^13^ Department of Pharmacology and Toxicology, College of Pharmacy, King Saud University, Riyadh, Saudi Arabia; ^14^ Department of Clinical Laboratory Sciences, College of Applied Medical Sciences, King Saud University, Riyadh, Saudi Arabia

**Keywords:** Khlalfa region, *Cannabis sativa*, KIF, Morocco, anti-inflammatory properties, burn, wound healing, toxicity

## Abstract

**Introduction:**

This study investigates the potential effects of cannabis seed oil (CSO) on the wound healing process. The aim was to assess the efficacy of CSO in treating skin wounds using an animal model and to explore its anti-inflammatory properties through in silico analysis.

**Methods:**

Eighteen male albino Wistar rats, weighing between 200 and 250 g, were divided into three groups: an untreated negative control group, a group treated with the reference drug silver sulfadiazine (SSD) (0.01 g/mL), and a group treated topically with CSO (0.962 g/mL). The initial wound diameter for all groups was 1 cm. In silico studies were conducted using Maestro 11.5 to evaluate the anti-inflammatory effects of phytoconstituents against cyclooxygenase-1 (COX-1) and cyclooxygenase-2 (COX-2).

**Results:**

CSO and SSD treatments led to a significant reduction (p <0.05) in the size of burned skin wounds by day 5, with contraction rates of 53.95% and 45.94%, respectively, compared to the untreated negative control group. By day 15, wounds treated with CSO and SSD had nearly healed, showing contraction rates of 98.8% and 98.15%, respectively. By day 20, the wounds treated with CSO had fully healed (100%), while those treated with SSD had almost completely healed, with a contraction rate of 98.97%. Histological examination revealed granulated tissue, neo-blood vessels, fibroblasts, and collagen fibers in wounds treated with CSO. In silico studies identified arachidic acid, γ-linolenic acid, and linolenic acid as potent inhibitors of COX-1 and COX-2. Serum biochemical parameters indicated no significant changes (p > 0.05) in liver and kidney function in rats treated with CSO, whereas a significant increase (p < 0.01) in ALAT level was observed in rats treated with SSD.

**Discussion:**

The findings demonstrate that CSO has a promising effect on wound healing. The CSO treatment resulted in significant wound contraction and histological improvements, with no adverse effects on liver and kidney function.However, the study's limitations, including the small sample size and the need for detailed elucidation of CSO's mechanism of action, suggest that further research is necessary. Future studies should focus on exploring the molecular pathways and signaling processes involved in CSO’s pharmacological effects.

## 1 Introduction

Marijuana, hashish, bhang, and others are the most common illicit drug preparations of C*annabis sativa* L. globally (Grinspoon et al., 1997). Two major phytoconstituents of *C. sativa* are Δ-9-tétrahydrocannabinol (THC) and cannabidiol (CBD) (Mechoulam et al., 1970; Murillo-Rodríguez et al., 2006). THC is known for its psychoactive activities, but CBD is not. While the stems and leaves of *C. sativa* contain these active molecules, another part, seeds, has captured our interest. Seeds have been found to have various health benefits. Because of their nutritional value and their role in lowering hypertension and cholesterol, seeds of *C. sativa* are beneficial to health (Jones, 1995). Seeds of *C. sativa* contain 20%–25% protein, 20%–30% hydrocarbons, 25%–35% oils, 10%–15% insoluble fiber, and minerals (Deferne and Pate, 1996; Pate, 1999). Cannabis seed oil (CSO) is a rich source of linoleic and α-linolenic fatty acids, with an optimum ratio (3:1) (Kriese et al., 2004) and thus well-balanced for human nutrition. CSO is rich in diverse bioactive constituents, including fatty acids, tocopherols, and sterols, that have functional properties (Stambouli et al., 2006). CSO contains γ-linolenic acid, known for its high penetration into the skin, so CSO can be used as an ideal compound in cosmetics for lipid-enriched creams (Rausch, 1995). At the experimental level, a study showed that when rats were fed 5% or 10% of cannabis seed meal containing CSO, functions of platelets were improved with a reduction in platelet aggregation (Richard et al., 2007) compared to a diet containing an equivalent amount of palm oil. Beneficial effects of CSO have also been observed during clinical studies. For instance, during a trial of 20 patients with eczema, dietary exposure to 30 mL CSO/day resulted in an increase in concentrations of essential fatty acids in blood compared to individuals fed olive oil (Callaway et al., 2005). Individuals receiving CSO also exhibited improvement in dry skin and ulceration compared to those consuming olive oil. These results were attributed to a balanced and abundant intake of polyunsaturated fatty acids in CSO.

Based on the previous observations of improved health of the integument of humans, we have focused on evaluating the effects of CSO on skin wounds, a health problem that adversely affects millions of people worldwide (Ract et al., 2015). Skin acts as an interface between internal organs and the external environment, forming a barrier that prevents the body’s dehydration and the penetration of external microorganisms (Pereira et al., 2013). Burned skin, or thermal injury, occurs when the skin faces extreme heat or fire, resulting in damage to its layers (Log, 2017; Martin and Falder, 2017). Initially, the outer layer, the epidermis, experiences immediate cell death due to heat, leading to blistering and redness as protective responses (Viner, 2018; Hussain et al., 2022). As the burn progresses deeper, it affects blood vessels, nerves, and other structures, causing inflammation and pain (Newell et al., 2020). Severe burns may extend into deeper layers, leading to tissue destruction and potential complications like infection and scarring (Kirwan and Pignataro, 2015). However, burned skin initiates a series of physiological responses aimed at repair and recovery (Guan et al., 2020). Inflammation, marked by increased blood flow and immune cell recruitment, aids in removing damaged cells and debris. Pain signals act protectively while the skin undergoes reepithelialization and collagen synthesis, facilitating wound closure and scar formation. Angiogenesis ensures adequate oxygen and nutrient supply for healing (Rose and Chan, 2016; Radzikowska-Büchner et al., 2023).

As cited below, this barrier has a natural ability to self-regenerate after damage, but this ability can be impaired when there is a significant loss of skin due to deep burns, chronic wounds, non-healing ulcers, or diabetes (Guo and DiPietro, 2010; Groeber et al., 2011). Despite several treatments by conventional medicine, therapeutic results often need to be improved. Hence, the use of herbal drugs can be explained by the perception that herbs can facilitate healing with minimal unwanted side effects. Specifically, CSO has captured our attention. This study was conducted to investigate the wound healing potential of CSO on burned skin wounds in rats. This study stands out from previous research due to its simplicity. Unlike other studies that often involve the use of additional materials such as dressing materials (films, hydrogels, etc.), this investigation focuses on the direct application of CSO to burned skin wounds. This direct application method distinguishes the study and allows for a more focused examination of the wound-healing potential of CSO.

The various components in cannabis seed oil may contribute to its potential in wound healing through diverse mechanisms. Unsaturated fatty acids such as α-linolenic acid and linoleic acid exhibit anti-inflammatory properties, which could aid in reducing inflammation at the wound site, thereby facilitating the initial stages of healing. Additionally, the moisturizing effects of these fatty acids help maintain a conducive environment for healing by preventing excessive dryness. Furthermore, antioxidants like tocopherols (vitamin E) in CSO can protect cells from oxidative stress, promoting cellular repair and regeneration.

## 2 Materials and methods

### 2.1 Plant material and drugs

Seeds of the Kif cultivar of cannabis were collected in the Khlalfa region, located in the northern part of Taounate, Morocco (34°39′ 36″ North, 4°36′ 36″ West). Plants were verified and authenticated by Pr. Bari Amina at the Laboratory of Biotechnology, Environment, Agro-Food, and Health at Sidi Mohamed Ben Abdellah University, and a specimen was placed in the herbarium of the Faculty of Sciences-Fez under the voucher number CS001kh1220. Seeds were washed with hexane to remove THC, and oil of *C. sativa* seeds (CSO) was obtained through cold pressing. Silver sulfadiazine (SSD) was procured from a local pharmacy to establish a reference drug.

### 2.2 Gas chromatography analysis

Fatty acids were transformed into methyl esters by transesterification with a solution of methanolic potassium hydroxide under reflux conditions, following the standardized procedure NF EN ISO 5509 (1). These methyl esters were identified and quantified by use of gas chromatography on a CARBOWAX 20 M (30 m × 0.25 mm x 0.25 μm) polar column. The gas chromatography-mass spectrometry (GCMS) system employed an Agilent autosystem chromatograph equipped with a split injector, operating at a temperature of 240°C with a split ratio of 30 and a mass spectrometry detector. The carrier gas was helium, at a rate of 1.5 mL/min, and the analyses were carried out using temperature programming, initiating at 140°C for 1 min, followed by a ramping of 15°C/min, and finally stabilizing at 200°C for 10 min (Ichihara and Fukubayashi, 2010).

### 2.3 Animals

Eighteen male albino Wistar rats with masses of 200–250 g were housed under laboratory conditions with a 12:12 h light–dark cycle at 22 ± 2°C. Rats were provided food and water *ad libitum*. Rats were divided into three groups of six individuals: 1) an untreated control, 2) a group treated with 0.2 mL of CSO per square centimeter at a concentration of 0.962 g/mL, and 3) a group treated with the reference drug SSD (0.01 g/mL).

### 2.4 Skin wound induction protocol

The skin wounds were generated following the technique outlined by Tavares Pereira et al. (2012), albeit with a minor modification (shaving the back of the animals was performed using a hair clipper rather than manual traction of hair, and the object used in creating skin burns was iron). Rats were anesthetized by administering pentobarbital anesthesia (50 mg/kg) via an intraperitoneal injection in the pharmacology laboratory situated within the sciences faculty at Sidi Mohamed Ben Abdellah University in Fez, Morocco. Then, a shaved region on the dorsal flank was cleansed with 70% ethanol, and a 1 cm diameter metal cylinder heated in boiling water was applied for 15 s. The wounds were photographed by a digital camera. Wounds were then treated topically with either CSO (0.962 g/mL) or SSD (0.01 g/mL) once daily in the morning (at 10:00 a.m.) until healing was complete. Wounds were measured using ImageJ Software, and the percentage of wound contraction was subsequently calculated (Equation 1). The methodologies employed in this investigation adhered to universally recognized guidelines for treating and utilizing laboratory animals, and consent was given by the Ethics Committee at the Faculty of Sciences, located in Fez, Morocco.

PWC=((Initialwoundsize−specificdaywoundsize)/(Initialwoundsize))*100 (1)

### 2.5 Histological analysis

Two rats from each treatment were euthanized 5 and 15 days post second-degree burn trauma. Burned tissues were collected, preserved in a 10% formalin solution, and stained with hematoxylin and eosin (H&E). Histological alterations were observed under a light microscope, and to visualize the presence of blood vessels, fibroblasts, and collagen fibers, photomicrographs were captured at 100× or 400× magnifications.

### 2.6 Molecular docking

This study used computational tools integrated into the Schrodinger Suites software (version 2018) for anti-inflammatory evaluation.

#### 2.6.1 Ligand preparation

All fatty acids identified in *C. sativa* seeds were considered in computational analysis. Ligands were adjusted to a theoretical pH of 7.0, and the ligprep panel within the Maestro 11.5 software was used. The OPLS3 force field was used with a tolerance of ±2.0. Desalting and tautomer generation methods were applied to optimize calculations while limiting the number of stereoisomer calculations to approximately thirty per ligand and using the output format specified by Maestro software (Aboul-Soud et al., 2022).

#### 2.6.2 Protein preparation

The crystal structures of cyclooxygenase-1 (COX-1) (Ayman et al., 2023) and cyclooxygenase-2 (COX-2) (Amrati et al., 2023a), identified by their PDB IDs of 3KK6 and 1PXX, respectively, were obtained from the RCSB database (http://www.rcsb.org/pdb) and imported into Maestro 11.8 workspaces. These proteins were processed using the Protein Production Wizard in the Schrodinger suite. During protein preprocessing, bond orders were assigned, water molecules were removed, and protonation states were adjusted to pH 7.0. Water molecules with fewer than three hydrogen bonds to non-water entities were eliminated. Protein minimization was performed using the OPLS3 force field (Amrati et al., 2023b).

#### 2.6.3 Receptor grid generation

To delineate the operational binding regions for ligand docking tasks, receptor grid files were created using the receptor grid formation panel, which involved the selection of relevant protein structures within the designated workspace. Subsequently, to forecast the extent of binding affinity and ascertain the nature of molecular interactions, compounds from *C. sativa* were interacted with active COX-1 and COX-2 sites (Aboul-Soud et al., 2022).

### 2.7 Sub-acute toxicity

At the termination of the study, the rats were euthanized, and blood was collected from the orbital sinus for use in biochemical analyses. Selected enzymes included alanine aminotransferase (ALAT), aspartate aminotransferase (ASAT), urea, and creatinine, which were analyzed through commercial kits.

### 2.8 Statistical analysis

Normality was checked using the Shapiro–Wilks test, whereas the assumption of homogeneity of variance was evaluated using Levene’s test. The values were presented as mean ± SEM. A two-way ANOVA statistical analysis was conducted to compare the control and treated groups, followed by the Tukey test, using GraphPad Prism 5 (Microsoft). A difference between the groups was deemed statistically significant if the *p*-value was less than 0.05.

## 3 Results and discussion

**FIGURE 1:**
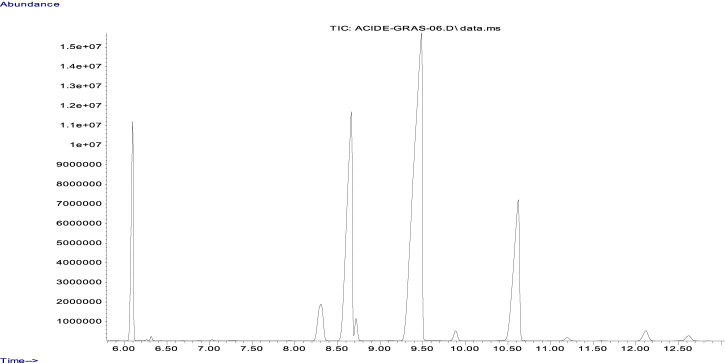
GC/MS chromatographic profile of the cannabis seed oil sample.

The fatty acid compositions expressed as a weight percentage of CSO have been defined previously. The major fatty acids and their relative proportions are linoleic acid C18:2 (46.6%), oleic acid C18:1 (22.32%), α-linolenic acid C18:3 (14.87%), palmitic acid C16:0 (8.83%), stearic acid C18:0 (3.5%), arachidic acid C20:0 (0.99%), arachidonic acid C20:1 (0.53%), γ-linolenic acid C18:3 (0.69%), and palmitoleic acid C16:1 (0.15%) (Figure 1; Table 1).

**TABLE 1:** Relative proportions of fatty acids in cannabis seed oil (CSO).

N°	Identification	%
1	Palmitic acid (C16:0)	8.83
2	Palmitoleic acid (C16:1)	0.15
3	Stearic acid (C18:0)	3.5
4	Oleic acid (C18:1)	22.32
5	Linoleic acid (C18:2)	46.6
6	γ-Linolenic acid (C18:3)	0.69
7	Linolenic acid (C18:3)	14.87
8	Arachidic acid (C20:0)	0.99
9	Arachidonic acid (C20:1)	0.53

**FIGURE 2:**
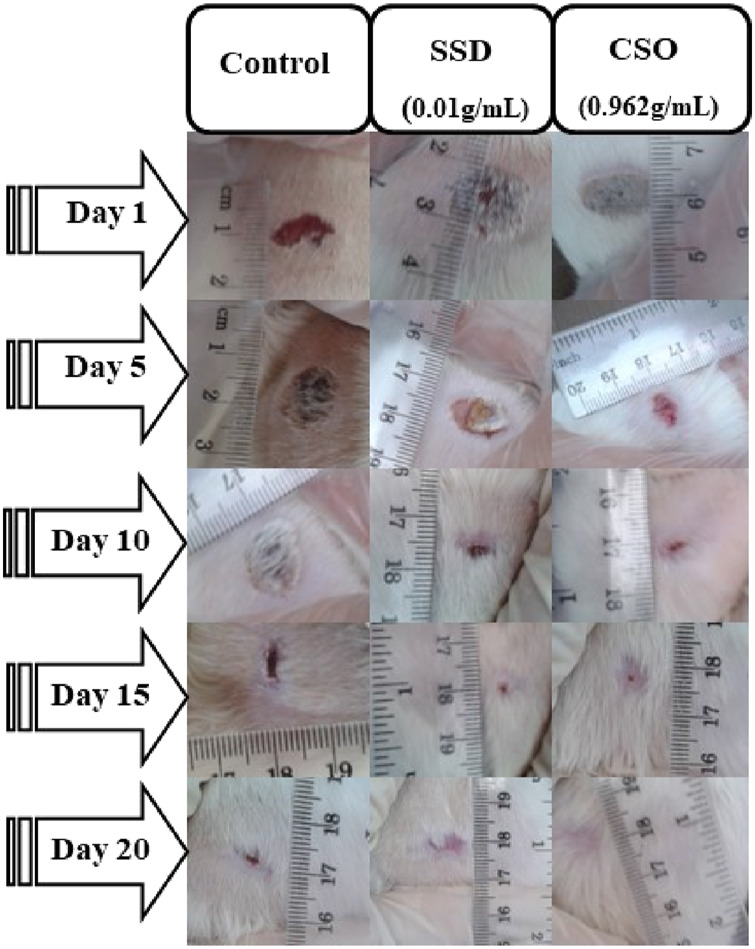
Macroscopic morphology of the burned skin wounds during different days of treatment.

No significant changes in the area of skin wounds (Figure 2) were observed during the first days following treatment. However, after 5 days, a significant decrease (*p* < 0.05) was noted in the area of skin wounds in the group treated with CSO compared to the negative control group. This result was also approximately similar to the one obtained using SSD. It has been reported that herbal drugs are widely used to treat skin wounds. *Cannabis sativa* L. seed oil was examined by testing the effects of its topical application in an experimental animal model of burned skin wounds using 18 male albino Wistar rats.

The size of burned skin wounds started to reduce significantly (*p* < 0.05) on day 5 (Table 2), with a percentage contraction of 53.95% for the group treated with CSO. Compared to the negative control group, wounds were approximately completely (98.8%) healed by day 15. This result demonstrated a slower healing process when rats were left untreated, with a percentage contraction area of 88.26% on day 20. Topical treatment with SSD significantly aided healing, as indicated by a greater percentage of contraction of the area of the wound. The size of skin wounds was significantly (*p* < 0.01) less than 10 days after the initial trauma, with a contraction area of 94.05% compared to the negative control, which had a reduction of 79.7%. In untreated rats, healing was not complete even 20 days post trauma, at which time the area of the burn had decreased by 88.26%.

**TABLE 2:** Effects of topical treatment with CSO (0.962 g/mL) or SSD (0.01 g/mL) on healing burned skin wounds.

Time (days)	Contraction of wounds (%)		
	Control	SSD (0.01 g/mL)	CSO (0.962 g/mL)
**1**	0	0	0
**5**	43.36 ± 3.34	45.94 ± 2.79	53.95 ± 2.71*
**10**	79.69 ± 2.49	94.05 ± 0.71##	92.54 ± 0.61**
**15**	83.61 ± 2.85	98.15 ± 0.51##	98.79 ± 0.19**
**20**	88.26 ± 1.58	98.97 ± 0.36##	100**

Data are expressed as mean ± SEM (n = 6). *p < 0.05, **p < 0.01 (vs. control), and ##*p* < 0.01 (vs. control). *: CSO, compared with control group; #: SSD, compared with control group.

**FIGURE 3:**
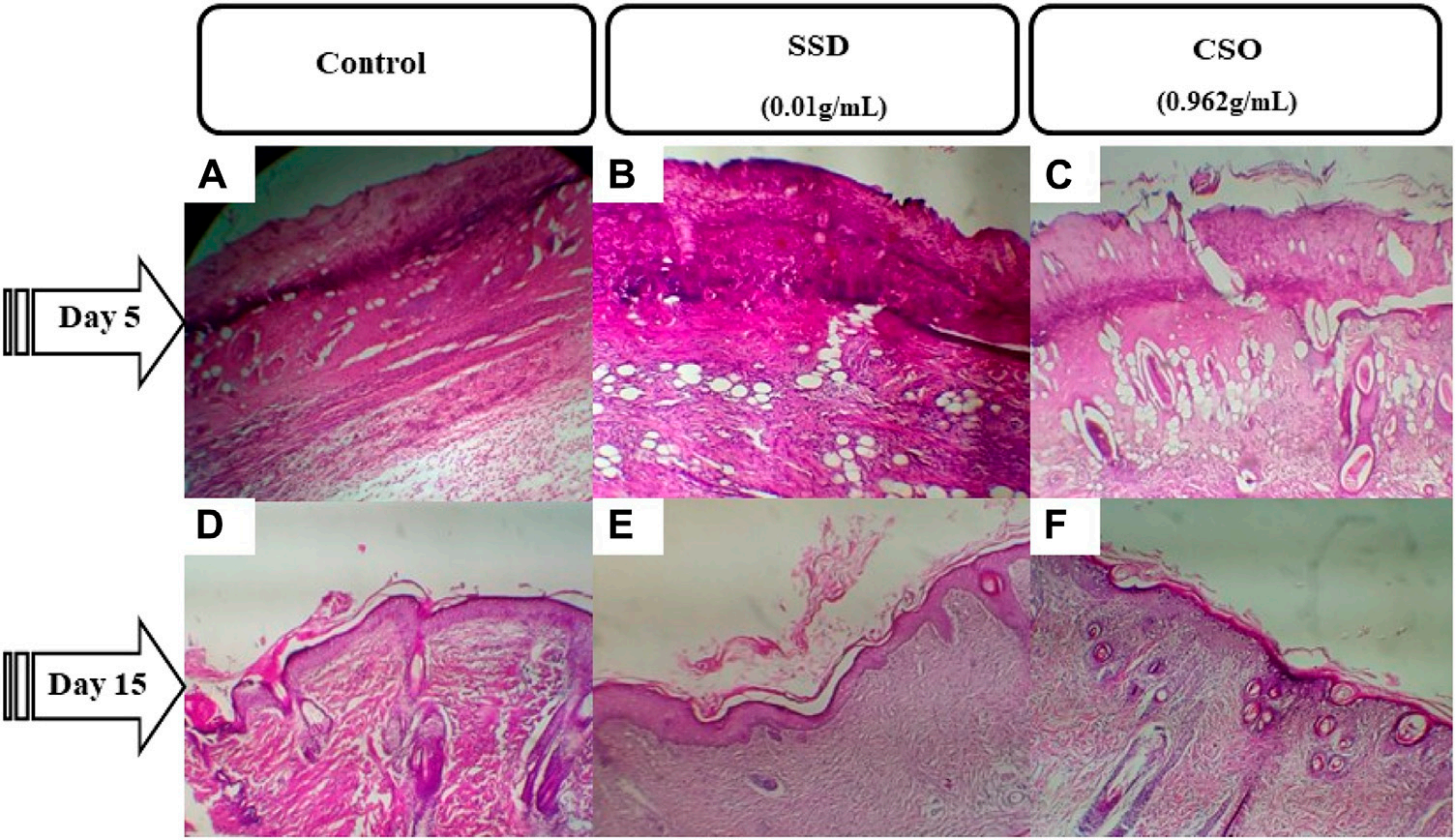
Photomicrographs of burned skin sections 5 days and 15 days after burns and various treatments. **(A)** untreated negative control on day 5, **(B)** group treated with SSD on day 5, and **(C)** group treated with CSO on day 5. **(D)** Untreated negative control group on day 15, **(E)** group treated with SSD on day 15, and **(F)** group treated with CSO on day 15. Images are acquired at a magnification of 100×.

On day 5, reepithelialization was not complete in all treatments (Figure 3). However, the burned area size was smaller, and inflammatory cell infiltration was less frequent in rats topically treated with CSO. By 15 days post trauma, reepithelialization, with better epithelium regeneration and regeneration of skin appendages, was well-developed in rats treated with CSO or SSD but not in the untreated, negative control rats.

Five days after injury, the burn surface area sections of rats treated with CSO were necrotic with slightly infiltrated dermal congestion (Figure 3. The derma was congested with increased neutrophils for the rats treated with SSD, the same as those of the negative control group (Figures 3A–C). Fifteen days post trauma in rats treated with CSO, granulation tissue clearly replaced the exudates (Figure 3F) with better epithelium regeneration and regeneration of skin appendages. Granulation tissue and epidermal regeneration were clear in rats treated with SSD. However, only a small amount of granulation tissue was observed in rats not treated with topical drugs.

**FIGURE 4:**
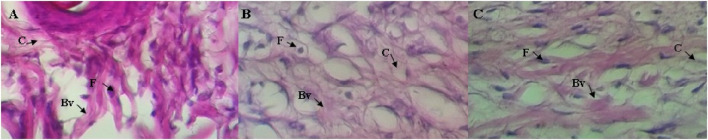
Histological investigations of the burn wound healing effects of CSO (0.962 g/mL) on rat’s skin: **(A)** negative control, **(B)** SSD (0.01 g/mL) treated group, and **(C)** group treated with CSO (0.962 g/mL) on day 15. The labeled arrows identify collagen (C), fibroblast (F), and a blood vessel (Bv). Images were acquired at a magnification of 400×.

Rats treated topically with CSO for 15 days had more blood vessels, fibroblasts, and collagen fibers in granulation tissues than the negative control rats (Figure 4C). The greater vascularization was similar to that caused by topical treatment with SSD (Figure 4B). Greater vascularization, more fibroblasts, and collagen production efficiently repair burn wounds. These results indicate that topical treatment with CSO accelerates the healing of skin burn wounds by keeping relatively more blood vessels, fibroblasts, and collagen fibers.

Sub-acute biochemical parameters, including alanine aminotransferase (ALAT), aspartate aminotransferase (ASAT), creatinine, and urea, are used as indicators for the evaluation of the toxic potential of drugs, including herbal products (Chebaibi et al., 2019; Musa et al., 2022; Jaber, 2023). ALAT and ASAT are enzymes primarily found in the liver, and elevated concentrations in blood plasma can indicate hepatocellular damage (Taj et al., 2019; Petrichev, 2021), a common side effect of some drugs. Creatinine and urea are indicators of kidney function (Ogbodo, 2021; Mamri et al., 2022). Elevated concentrations are indicative of impaired renal function, which can be caused by drug-induced nephrotoxicity. The safety and efficacy of pharmaceutical compounds are assessed by monitoring these parameters during drug trials and clinical use. These parameters are all indicators of potential adverse effects on vital organs, so evaluation of these biochemical markers is useful for identifying and managing drug-induced toxicities, ultimately contributing to safer drug development and patient care. No significant changes in ASAT, creatinine, or urea (*p* > 0.05) were observed among the treatments, including the negative control group, the SSD group, and the CSO group (Table 3), which indicated no adverse effects on either liver or kidney function. These findings are supported by previous studies (Hartsel et al., 2019; Chelminiak-Dudkiewicz et al., 2022). However, significantly (*p* < 0.01) greater concentrations of ALAT were observed in rats treated with SSD than in the negative control rats. In fact, multiple controlled experiments and clinical studies have indicated that SSD could be toxic to the liver and other organs (Goldenheim, 1993; Fuller, 2009; Brandt et al., 2012; Musa and Elnabi, 2015).

**TABLE 3:** Biochemical parameters of rats treated with topical CSO or SSD.

	Negative control	SSD (0.01 g/mL)	CSO (0.962 g/mL)
ALT (IU/L)	55.66 ± 3.53	81.86 ± 3.43^a^	71.76 ± 4.39
AST (IU/L)	142.17 ± 6.44	166.65 ± 6.15	163.84 ± 7.55
Creatinine (mg/L)	3.84 ± 0.11	4.25 ± 0.50	3.42 ± 0.44
Urea (g/L)	0.384 ± 0.03	0.416 ± 0.011	0.397 ± 0.050

Values are represented as mean ± SEM, n = 3.

^a^ Significant difference between SSD (0.01 g/mL) and the negative control group (*p* < 0.01).

Overall, these results show enhanced healing after topical treatment with CSO, compared to the untreated, negative control rats. This effect on healing was approximately comparable to that effected by topical treatment with SSD. The healing effects of CSO observed during this study could be attributed to physicochemical properties that provide a barrier, thus enhancing healing. In addition, CSO exerts anti-inflammatory effects (Musa et al., 2022), which facilitate healing. Due to its hydrophobic properties and lipid constituents, CSO can protect burns from drying and thus promote healing, a property that is widely exploited in cosmetology. SSD is used for its antimicrobial effect to inhibit infection, which promotes the healing of wounds (Groeber et al., 2011). SSD is widely used for the treatment of wounds mainly due to its wide spectrum of activity, ease of application, and minimal pain (Ichihara and Fukubayashi, 2010). Despite these benefits, several adverse effects of SSD, such as cases of renal toxicity and leukopenia, the emergence of resistant strains of microbial species, cases of delayed healing, and allergic reactions to silver, have been reported and might limit the use of SSD in some patients (Hollinger, 1996; Tavares Pereira et al., 2012; Aboul-Soud et al., 2022; Ayman et al., 2023).

Cyclooxygenase-1 (COX-1) and cyclooxygenase-2 (COX-2) are central players in synthesizing prostaglandins, lipids that are potent mediators of inflammation, pain, and fever. COX-1 is constitutively expressed and contributes to normal physiological functions, including protection of the gastric mucosal lining and regulation of blood platelets. COX-2 is inducible and is upregulated during inflammation, serving as a key contributor to the inflammatory response. By selectively or non-selectively targeting these enzymes through medications like NSAIDs or selective COX-2 inhibitors, it is possible to alleviate inflammation-related symptoms and improve the quality of life for individuals suffering from various inflammatory conditions.

**FIGURE 5:**
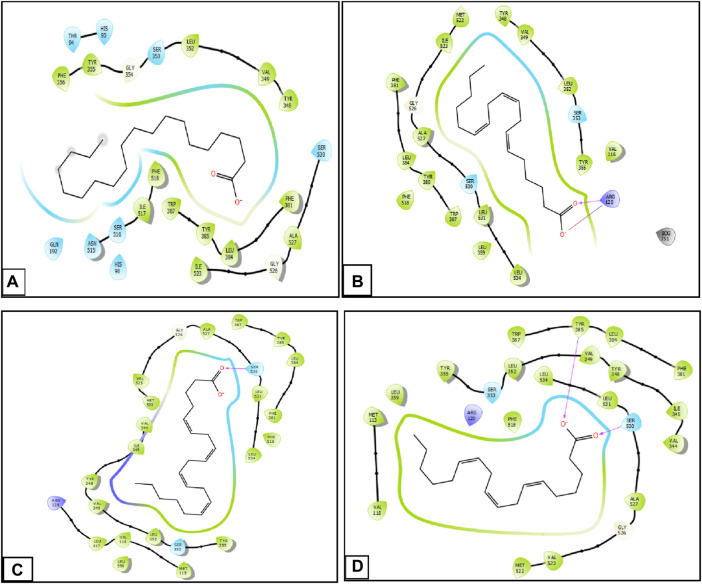
Two-dimensional view of ligands in active sites. **(A)** Arachidic acid interactions in the active sites of COX-1. **(B and D)** γ -Linolenic acid interactions in the active sites of COX-1 and COX-2. **(C)** Arachidonic acid interactions in the active sites of COX-2.

**FIGURE 6:**
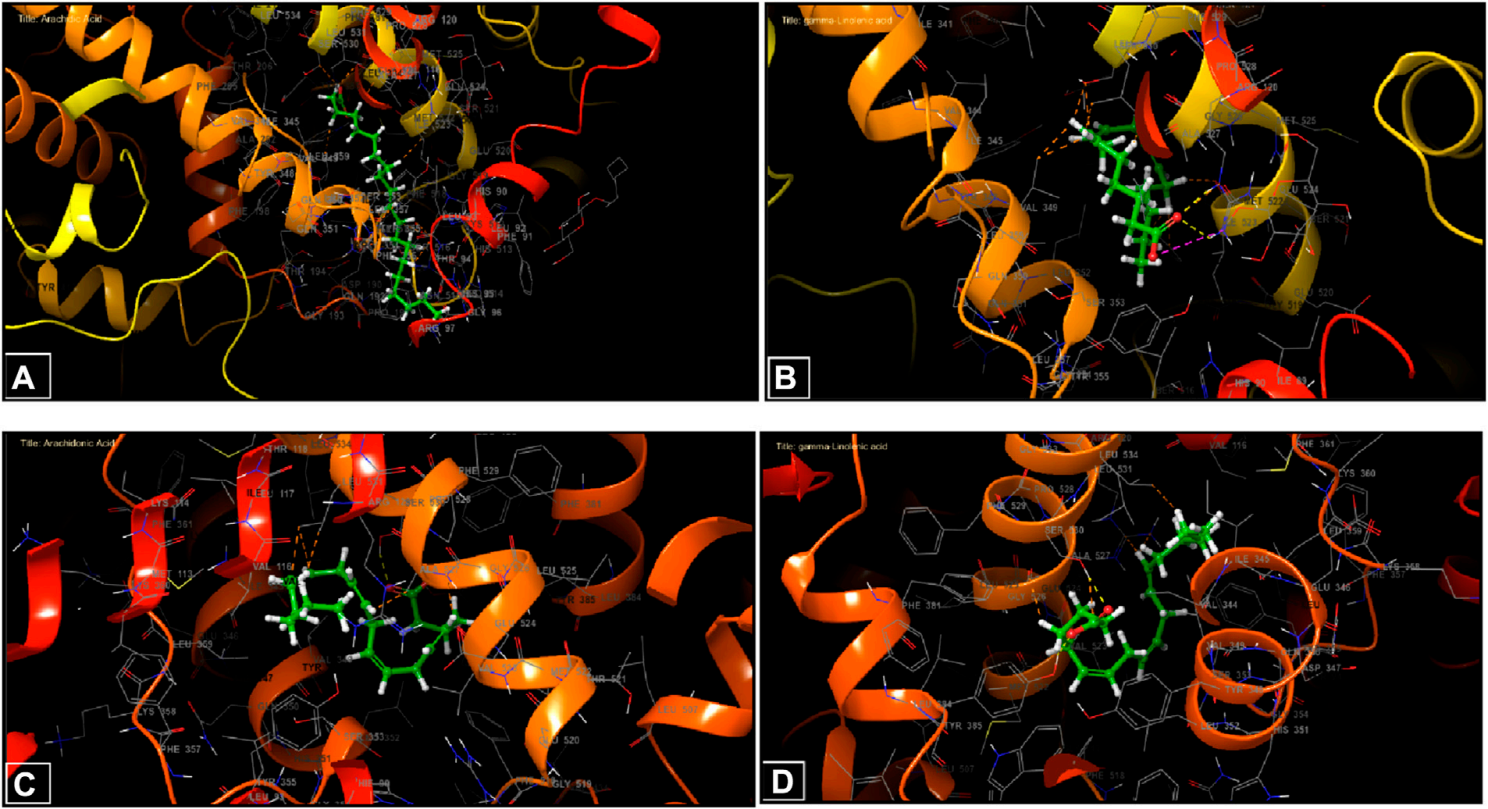
Three-dimensional view of ligands in active sites. **(A)** Arachidic acid interactions in the active sites of COX-1. **(B and D)** γ-Linolenic acid interactions in the active sites of COX-1 and COX-2. **(C)** Arachidonic acid interactions in the active sites of COX-2.

During inhibition of cycloxygenase 1, arachidic acid, γ-linolenic acid, and linolenic acid presented the greatest inhibitory energy with glide scores of −4.298 kcal/mol, −3.661 kcal/mol, and −2.486 kcal/mol, respectively (Table 4). Docking of γ-linolenic acid in the active site of COX-1 suggested the formation of a single hydrogen bond and a salt bridge with the residue ARG 120 (Figure 5B; Figure 6B). Furthermore, arachidonic acid, γ-linolenic acid, and linolenic acid were the most potent molecules against the active site of COX-2, with glide scores of −6.842 kcal/mol, −4.143 kcal/mol, and −3.252 kcal/mol, respectively (Table 4). Arachidonic acid formed a single bond with residue SER 530 in the active site of COX-2, while γ-linolenic acid established two hydrogen bonds with residues SER 530 and TYR 385 in the same active site (Figures 5C,D, 6C,D).

**TABLE 4:** Docking results with fatty acids identified in *Cannabis sativa* in the active sites of COX-1 and COX-2.

Title	COX-1 (PDB: 3KK6)			COX-2 (PDB:1PXX)		
	Glide gscore (kcal/mol)	Glide emodel (kcal/mol)	Glide energy (kcal/mol)	Glide gscore (kcal/mol)	Glide emodel (kcal/mol)	Glide energy
Arachidonic acid	-	-	-	−6.842	−22.996	−26.359
Arachidic acid	−4.298	−33.306	−27.968	-	-	-
γ-Linolenic acid	−3.661	−36.095	−30.637	−4.143	−41.25	−35.174
Linolenic acid	−2.486	−16.308	−23.148	−3.252	−35.728	−33.251
Palmitoleic acid	−2.419	−33.499	−32.888	−2.413	−33.252	−29.198
Linoleic acid	−2.269	−17.326	−23.199	−2.529	−30.248	−29.313
Oleic acid	−1.926	−24.995	−26.027	−3.614	−33.376	−33.601
Palmitic acid	−1.793	−27.309	−26.65	−2.509	−29.59	−28.931
Stearic acid	−0.898	−29.91	−32.554	−1.264	−22.467	−23.316

To exceed some limitations of use, several natural alternatives to SSD have been proposed that exhibited comparable or even better results than those of SSD. It has been reported that *Crocus sativus* is significantly better than SSD, resulting in wounds with fewer inflammatory cells and an epidermis more similar to the normal structure. A comparative study between the healing effect of *Aloe vera* and SSD performed by Hosseinimehr et al. (2010) demonstrated that *A. vera* exhibited better wound reepithelialization than that induced by topical treatment with SSD.

Like other natural products, the effects of CSO on the healing of burn wounds might be attributable to the various phytochemical constituents that make up its composition. Natural healing products manifest their effects through one of the following mechanisms: antimicrobial, anti-inflammatory, antioxidant, stimulation of the skin collagen synthesis, cell proliferation, and/or angiogenic effects (Trop et al., 2006). The chemical composition of CSO, such as fatty acids, tocopherols, and phytosterols (Stambouli et al., 2006), could explain the facilitation of healing. Constituents of CSO can act through various mechanisms involving barrier and protective, antioxidant, and anti-inflammatory effects. CSO contains tocopherols, such as α-tocopherol (vitamin E), which has significant biological activities, including being an antioxidant (Fuller, 2009), which can minimize the oxidative stress that occurs during wounding. Topical treatment with tocopherols has also been shown to improve the healing process of excisional wounds in diabetic rats (Brandt et al., 2012). In addition, oleic and linoleic fatty acids (Musa and Elnabi, 2015) might also be antioxidants. The results of some studies have indicated that these acids can affect neutrophils, which are a source of free radicals in wounds (Goldenheim, 1993; McNulty et al., 2004). CSO also contains sterols, such as β-sitosterol, and studies have demonstrated that in addition to its efficacy in lowering hypercholesterolemia, it has antifungal, antiviral, and anti-inflammatory properties (Atiyeh et al., 2007). Despite limited data from studies focused on the use of CSO for treating skin burns, available evidence generally supports the oil’s positive effects on wound healing. Research suggests that CSO has a balanced ratio of ω-3 and ω-6 fatty acids, which are crucial for reducing inflammation and promoting tissue regeneration (Smith, 2000; Komarnytsky et al., 2021; Ben Necib et al., 2022). Additionally, the oil is rich in antioxidants such as vitamin E, known for their skin-repairing properties (Raiciu et al., 2016; Kotnala et al., 2019; Bakowska-Barczak et al., 2022). These characteristics are believed to improve skin elasticity and reduce scarring during the healing process. A similar report on the wound-healing efficacy of CSO has been noted in the literature (El Ghacham et al., 2023). The *in silico* studies could complement this report, providing additional insights into the potential mechanisms through which CSO promotes healing. While more rigorous clinical trials are needed to substantiate these findings, consistent reports of its therapeutic benefits in wound healing indicate that cannabis seed oil could be a valuable addition to skincare regimens designed for wound healing and recovery.

## 4 Conclusion

The results of the present study demonstrated the efficacy of CSO in the healing of burn wounds in rats, indicating it might represent a natural compound that can be used to treat injuries to the skin in humans and animals. CSO can also be an excellent drug in the cosmetic field because of its potential protection against skin problems via its efficacy in cicatrization. The results presented here provide the basis for future clinical studies demonstrating the safety and efficacy of CSO as a topical agent to facilitate the healing of wounds in humans. Finally, these findings underscore the safety profile of CSO as a natural product compared to the reference control group. These robust data further support the promising potential of CSO as a safe and viable option for dermal applications, with no adverse effects observed on liver and kidney functions. While the current study sheds light on the beneficial effects of CSO on wound healing, several avenues remain for further exploration. Future research endeavors could focus on conducting immuno-histocompatibility tests to better understand the immunological response to CSO. Additionally, investigating the long-term effects of CSO consumption on kidney and liver function, cardiovascular health, etc., would provide valuable insights into its safety profile. Furthermore, exploring the potential synergistic effects of CSO in combination with other natural compounds could lead to the development of more effective therapeutic interventions in other pharmacological investigations. Finally, elucidating the precise mechanisms underlying the therapeutic actions of CSO at the molecular level through advanced techniques such as proteomics and metabolomics would enhance our understanding and facilitate targeted drug development.

## Data Availability

The original contributions presented in the study are included in the article/Supplementary Material; further inquiries can be directed to the corresponding authors.

## References

[B1] Aboul-Soud M. A. Ennaji H. Kumar A. Alfhili M. A. Bari A. Ahamed M. (2022). Antioxidant, anti-proliferative activity and chemical fingerprinting of centaurea calcitrapa against breast cancer cells and molecular docking of caspase-3. Antioxidants 11 (8), 1514. 10.3390/antiox11081514 36009233 PMC9405406

[B2] Amrati F.E.-Z. Chebaibi M. Galvão de Azevedo R. Conte R. Slighoua M. Mssillou I. (2023a). Phenolic composition, wound healing, antinociceptive, and anticancer effects of caralluma europaea extracts. Molecules 28 (4), 1780. 10.3390/molecules28041780 36838767 PMC9961855

[B3] Amrati F.E.-Z. Elmadbouh O. H. M. Chebaibi M. Soufi B. Conte R. Slighoua M. (2023b). Evaluation of the toxicity of Caralluma europaea (CE) extracts and their effects on apoptosis and chemoresistance in pancreatic cancer cells. J. Biomol. Struct. Dyn. 41 (17), 8517–8534. 10.1080/07391102.2022.2135595 36271642

[B4] Atiyeh B. S. Costagliola M. Hayek S. N. Dibo S. A. (2007). Effect of silver on burn wound infection control and healing: review of the literature. burns 33 (2), 139–148. 10.1016/j.burns.2006.06.010 17137719

[B5] Ayman R. Radwan A. M. Elmetwally A. M. Ammar Y. A. Ragab A. (2023). Discovery of novel pyrazole and pyrazolo [1, 5‐a] pyrimidine derivatives as cyclooxygenase inhibitors (COX‐1 and COX‐2) using molecular modeling simulation. Arch. Pharm. 356 (2), 2200395. 10.1002/ardp.202200395 36336646

[B6] Bakowska-Barczak A. (2022). “Industrial hemp-based dietary supplements and cosmetic products,” in Industrial hemp (Germany: Elsevier), 247–299.

[B7] Ben Necib R. Manca C. Lacroix S. Martin C. Flamand N. Di Marzo V. (2022). Hemp seed significantly modulates the endocannabinoidome and produces beneficial metabolic effects with improved intestinal barrier function and decreased inflammation in mice under a high-fat, high-sucrose diet as compared with linseed. Front. Immunol. 13, 882455. 10.3389/fimmu.2022.882455 36238310 PMC9552265

[B8] Brandt O. Mildner M. Egger A. E. Groessl M. Rix U. Posch M. (2012). Nanoscalic silver possesses broad-spectrum antimicrobial activities and exhibits fewer toxicological side effects than silver sulfadiazine. Nanomedicine Nanotechnol. Biol. Med. 8 (4), 478–488. 10.1016/j.nano.2011.07.005 21839058

[B9] Callaway J. Schwab U. Harvima I. Halonen P. Mykkänen O. Hyvönen P. (2005). Efficacy of dietary hempseed oil in patients with atopic dermatitis. J. Dermatological Treat. 16 (2), 87–94. 10.1080/09546630510035832 16019622

[B10] Chebaibi M. Bousta D. Chbani L. Ez zoubi Y. Touiti N. Achour S. (2019). Acute toxicity of plants mixture used in traditional treatment of edema and colic renal in Morocco. Sci. Afr. 6, e00152. 10.1016/j.sciaf.2019.e00152

[B11] Chelminiak-Dudkiewicz D. Smolarkiewicz-Wyczachowski A. Mylkie K. Wujak M. Mlynarczyk D. T. Nowak P. (2022). Chitosan-based films with cannabis oil as a base material for wound dressing application. Sci. Rep. 12 (1), 18658. 10.1038/s41598-022-23506-0 36333591 PMC9636169

[B12] Deferne J.-L. Pate D. W. (1996). International hemp association. J. Int. Hemp Assoc. 3 (1).

[B13] El Ghacham S. Bakali I. E. Zarouki M. A. Ali Y. A. E. H. Ismaili R. Ayadi A. E. (2023). Wound healing efficacy of Cannabis sativa L. essential oil in a mouse incisional wound model: a possible link with stress and anxiety. South Afr. J. Bot. 163, 488–496. 10.1016/j.sajb.2023.11.005

[B14] Fuller F. W. (2009). The side effects of silver sulfadiazine. J. burn care & Res. 30 (3), 464–470. 10.1097/bcr.0b013e3181a28c9b 19349889

[B15] Goldenheim P. (1993). An appraisal of povidone-iodine and wound healing. Postgrad. Med. J. 69, S97–S105. 8290466

[B16] Grinspoon L. Bakalar J. B. Zimmer L. Morgan J. P. (1997). Marijuana addiction. Science 277 (5327), 749–753. 10.1126/science.277.5327.749a 9273692

[B17] Groeber F. Holeiter M. Hampel M. Hinderer S. Schenke-Layland K. (2011). Skin tissue engineering—*in vivo* and *in vitro* applications. Adv. drug Deliv. Rev. 63 (4-5), 352–366. 10.1016/j.addr.2011.01.005 21241756

[B18] Guan Y. Sun F. Zhang X. Peng Z. jiang B. Liang M. (2020). Silk fibroin hydrogel promote burn wound healing through regulating TLN1 expression and affecting cell adhesion and migration. J. Mater. Sci. Mater. Med. 31, 48–11. 10.1007/s10856-020-06384-8 32405818

[B19] Guo S. a. DiPietro L. A. (2010). Factors affecting wound healing. J. Dent. Res. 89 (3), 219–229. 10.1177/0022034509359125 20139336 PMC2903966

[B20] Hartsel J. A. Boyar K. Pham A. Silver R. J. Makriyannis A. (2019). Cannabis in veterinary medicine: cannabinoid therapies for animals. Nutraceuticals veterinary Med., 121–155. 10.1007/978-3-030-04624-8_10

[B21] Hollinger M. A. (1996). Toxicological aspects of topical silver pharmaceuticals. Crit. Rev. Toxicol. 26 (3), 255–260. 10.3109/10408449609012524 8726163

[B55] Hosseinimehr S. J. Khorasani G. Azadbakht M. Zamani P. Ghasemi M. Ahmadi A. (2010). Effect of aloe cream versus silver sulfadiazine for healing burn wounds in rats. Acta Dermatovenerologica Croatica 18 (1). 20361881

[B22] Hussain Z. Thu H. E. Rawas-Qalaji M. Naseem M. Khan S. Sohail M. (2022). Recent developments and advanced strategies for promoting burn wound healing. J. Drug Deliv. Sci. Technol. 68, 103092. 10.1016/j.jddst.2022.103092

[B23] Ichihara K. i. Fukubayashi Y. (2010). Preparation of fatty acid methyl esters for gas-liquid chromatography. J. lipid Res. 51 (3), 635–640. 10.1194/jlr.d001065 19759389 PMC2817593

[B24] Jaber S. A. (2023). *In vitro* alpha-amylase and alpha-glucosidase inhibitory activity and *in vivo* antidiabetic activity of Quercus coccifera (Oak tree) leaves extracts. Saudi J. Biol. Sci. 30 (7), 103688. 10.1016/j.sjbs.2023.103688 37292253 PMC10245109

[B25] Jones K. (1995). Nutritional and medicinal guide to hemp seed. Rainfor. Bot. Lab.

[B26] Kirwan H. Pignataro R. (2015). The skin and wound healing. Pathology Intervention Musculoskelet. Rehabilitation 25, 1352–1356.

[B27] Komarnytsky S. Rathinasabapathy T. Wagner C. Metzger B. Carlisle C. Panda C. (2021). Endocannabinoid system and its regulation by polyunsaturated fatty acids and full spectrum hemp oils. Int. J. Mol. Sci. 22 (11), 5479. 10.3390/ijms22115479 34067450 PMC8196941

[B28] Kotnala A. (2019). Indian Medicinal Plants for skin care and cosmeceuticals: a review. J. Biomed. Ther. Sci. 6 (2), 24–60.

[B29] Kriese U. Schumann E. Weber W. Beyer M. Brühl L. Matthäus B. (2004). Oil content, tocopherol composition and fatty acid patterns of the seeds of 51 Cannabis sativa L. genotypes. Euphytica 137, 339–351. 10.1023/b:euph.0000040473.23941.76

[B30] Log T. (2017). Modeling skin injury from hot spills on clothing. Int. J. Environ. Res. public health 14 (11), 1374. 10.3390/ijerph14111374 29137118 PMC5708013

[B31] Mamri S. Daoudi N. E. Marghich M. Ouahhoud S. Khoulati A. Choukri M. (2022). Protective effect of Crocus sativus stamens extract on gentamicin-induced nephrotoxicity and oxidative damage in rat kidney. J. Exp. Biol. Agric. Sci. 10 (1), 73–82. 10.18006/2022.10(1).73.82

[B32] Martin N. Falder S. (2017). A review of the evidence for threshold of burn injury. Burns 43 (8), 1624–1639. 10.1016/j.burns.2017.04.003 28536038

[B33] McNulty C. Rodgers G. L. Mortensen J. E. (2004). An overview of the topical antimicrobial agents used in the treatment of burn wounds. J. Continuing Educ. Top. Issues 273, 74–78.

[B34] Mechoulam R. Shani A. Edery H. Grunfeld Y. (1970). Chemical basis of hashish activity. Science 169 (3945), 611–612. 10.1126/science.169.3945.611 4987683

[B35] Murillo-Rodríguez E. Millán-Aldaco D. Palomero-Rivero M. Mechoulam R. Drucker-Colín R. (2006). Cannabidiol, a constituent of Cannabis sativa, modulates sleep in rats. FEBS Lett. 580 (18), 4337–4345. 10.1016/j.febslet.2006.04.102 16844117

[B36] Musa A. H. Hagos A. D. Dimsu G. G. Eshetu E. M. Tola M. A. Admas A. (2022). Subchronic toxicity study of herbal tea of Moringa stenopetala (Baker f.) Cudof. and Mentha spicata L. leaves formulation in Wistar albino rats. Toxicol. Rep. 9, 797–805. 10.1016/j.toxrep.2022.03.043 36518443 PMC9742831

[B37] Musa E. Elnabi E. B. S. J. (2015). Anti-inflammatory activity of the plant Cannabis sativa (L) petrolium ether extract in albino rats. Res. Pharm. 1 (3).

[B38] Newell C. F. Edwards F. J. Winograd S. M. (2020). A review of thermal burns for emergency clinicians. Emerg. Med. Rep. 41 (12).

[B39] Ogbodo E. (2021). Kidney function status in persons occupationally exposed to heavy metals in metal forging factory in nnewi, southeastern Nigeria. Afr. J. Biomed. Res. 24 (1), 151–157.

[B40] Pate D. W. (1999). Hemp seed: a valuable food source. Adv. hemp Res., 243–255.

[B41] Pereira R. F. Barrias C. C. Granja P. L. Bartolo P. J. (2013). Advanced biofabrication strategies for skin regeneration and repair. Nanomedicine 8 (4), 603–621. 10.2217/nnm.13.50 23560411

[B42] Petrichev M. (2021). Some important biochemical parameters in clinical veterinary toxicology. Tradition Mod. Veterinary Med. 6 (2).

[B43] Ract J. N. R. Soares F. A. S. D. M. Rodrigues H. G. Bortolon J. R. Murata G. M. Gonçalves M. I. A. (2015). Production of vegetable oil blends and structured lipids and their effect on wound healing. Braz. J. Pharm. Sci. 51, 415–427. 10.1590/s1984-82502015000200019

[B44] Radzikowska-Büchner E. Łopuszyńska I. Flieger W. Tobiasz M. Maciejewski R. Flieger J. (2023). An overview of recent developments in the management of burn injuries. Int. J. Mol. Sci. 24 (22), 16357. 10.3390/ijms242216357 38003548 PMC10671630

[B45] Raiciu A. D. (2016). Therapeutic applications of vegetable oils and GC-MS evaluation of ω-3, ω-6 and ω-9 amounts in six oleaginous plants. Rev. Chim. 67, 2449–2453.

[B46] Rausch P. (1995). Verwendung von hanfsamenol in der kosmetik. Cologne, Germany: Bioresourcehemp. Nova-Institute.

[B47] Richard M. Ganguly R. Steigerwald S. Al‐khalifa A. Pierce G. (2007). Dietary hempseed reduces platelet aggregation. J. Thrombosis Haemostasis 5 (2), 424–425. 10.1111/j.1538-7836.2007.02327.x 17155962

[B48] Rose L. F. Chan R. K. (2016). The burn wound microenvironment. Adv. wound care 5 (3), 106–118. 10.1089/wound.2014.0536 PMC4779284 26989577

[B49] Smith K. (2000). Hempseed oil: a smart start. Hemp Rep. 2 (14), 1488–3988.

[B50] Stambouli H. (2006). “Caractérisation de l’huile de graines de Cannabis sativa L. cultivé au nord du Maroc,” in Annales de Toxicologie Analytique (China: EDP Sciences).

[B51] Taj D. Tariq A. Sultana V. Ara J. Ahmad V. U. Ehteshamul-Haque S. (2019). Protective role of Stokeyia indica in liver dysfunction and associated complications in acetaminophen intoxicated rats. Clin. Phytoscience 5 (1), 28–8. 10.1186/s40816-019-0122-2

[B52] Tavares Pereira D. d.S. Lima-Ribeiro M. H. M. de Pontes-Filho N. T. Carneiro-Leão A. M. d. A. Correia M. T. d. S. (2012). Development of animal model for studying deep second-degree thermal burns. BioMed Res. Int. 2012, 1–7. 10.1155/2012/460841 PMC3379528 22736951

[B53] Trop M. Novak M. Rodl S. Hellbom B. Kroell W. Goessler W. (2006). Silver-coated dressing acticoat caused raised liver enzymes and argyria-like symptoms in burn patient. J. Trauma Acute Care Surg. 60 (3), 648–652. 10.1097/01.ta.0000208126.22089.b6 16531870

[B54] Viner T. C. (2018). Thermal/electrical injuries. Veterinary Forensic Pathol. 2, 17–35. 10.1007/978-3-319-67175-8_2

